# Migration of Melamine and Its Derivatives from Melamine/Bamboo/Wheat Straw-Made Tableware Purchased from Internet Markets or Retail Shops in China

**DOI:** 10.3390/toxics12020143

**Published:** 2024-02-09

**Authors:** Shaojie Liu, Yifei Wang, Zhanren Liu, Zhiping Yang, Liang Chen, Bo Chen

**Affiliations:** 1 School of Public Health, Fujian Medical University, Fuzhou 350122, China; liushaojie@fudan.edu.cn; 2 School of Public Health, Key Laboratory of Public Health Safety of the Ministry of Education, Fudan University, Shanghai 200032, China; 22111020064@m.fudan.edu.cn (Y.W.); 23111020068@m.fudan.edu.cn (Z.L.); 3 Department of Clinical Nutrition, The First Affiliated Hospital of Xiamen University, School of Medicine, Xiamen University, Xiamen 361003, China; zhipingyang2020@163.com; 4 College of Biological & Environmental Sciences, Zhejiang Wanli University, Ningbo 315100, China

**Keywords:** melamine and its derivatives, food contact materials, migration, exposure assessment

## Abstract

Objectives: The ecofriendly and sustainable concept of bamboo- and wheat straw-made tableware has gained attention in recent years. However, it is necessary to note that these kinds of tableware are composed of melamine (MEL)–formaldehyde resin with the addition of bamboo fibers or wheat straw. This study aims to explore the potential migration of MEL and its derivatives from the tableware and conduct a risk assessment. Methods: The study involved 46 bowls or cups purchased from Internet markets or retail shops in China, whose raw materials included MEL, bamboo, and wheat straw. There were four pieces of glass- or ceramic-made tableware used as the control group. Migration testing was performed according to the test conditions selected from the European Union Reference Laboratory for Food Contact Materials. Considering the realistic worst-case scenario, we measured the concentrations of MEL and its derivatives in food simulants using ultra-performance liquid chromatography–tandem mass spectrometry and estimated the exposure risks for adults and 1-year-old infants. Results: MEL and its derivatives could migrate from MEL-, bamboo-, and wheat straw-made tableware with varying concentrations. The total migration was ranked as follows: bamboo-made tableware > MEL-made tableware > wheat straw-made tableware > glass- or ceramic-made tableware (*p* < 0.001). The primary contributor to the total concentration for MEL- and bamboo-made tableware was MEL, whereas cyanuric acid (CYA) was the main contributor for wheat straw-made tableware. Based on the total concentration of MEL and its derivatives and the strictest TDI value, the proportions of the calculated hazard quotient ≥1 for MEL-, bamboo-, and wheat straw-made tableware in adults were 53.50%, 92.30%, and 1.90%; and the proportions in 1-year-old infants increased to 86.00%, 100.00%, and 7.40%. Conclusion: The utilization of MEL-, bamboo-, and wheat straw-made tableware could be regarded as a significant source of human exposure to MEL and its derivatives. It is advisable for both adults and infants to refrain from using tableware manufactured with MEL and bamboo fiber, as it may increase the susceptibility to MEL-related diseases.

## 1. Introduction

Melamine (MEL) is a triazine compound with nitrogenous heterocycles, which has raised human concerns due to its involvement in illegal adulteration scandals, such as the pet food recall of 2007 in America and the milk scandal of 2008 in China [1,2]. The occurrence of illegal adulteration under the continuous supervision of national governments has become increasingly rare, giving rise to other scenarios such as food contamination. The World Health Organization (WHO) has announced that food consumption is the primary source of human exposure to MEL in daily life [3]. This includes foods themselves and the migration of MEL from MEL–formaldehyde resin-made tableware [4,5]. Based on the previous literature, the concentration of MEL migrating from MEL-made tableware was found to be higher than that presented in foods themselves [6,7]. Therefore, increased attention should be directed towards the migration of MEL from tableware manufactured with MEL. MEL-made tableware is produced through the polymerization reaction of melamine and formaldehyde. Owing to its outstanding wear resistance, impact resistance, impressive extensibility, and cost-effectiveness, MEL-made tableware has gained widespread popularity among both restaurants and households [8,9,10]. Moreover, it exhibits excellent versatility in terms of color, shape, and design, making it highly appealing to infants [11]. However, MEL will migrate persistently from MEL-made tableware when loading foods, especially in high-temperature and high-acid environments [7,12,13]. Thus, the risk assessment of exposure to MEL through contact with MEL-made tableware is essential for both adults and infants. In addition, cyanuric acid (CYA) is a common derivative of MEL and is generally used in synthetic adhesives. It may be added to the production of MEL-made tableware for bonding and forming, which will migrate with MEL. However, the majority of previous studies mainly come from abroad and report on the migration of a single substance, MEL, from MEL-made tableware [7,12,13]. Few studies have been performed in China, and these studies have focused on the possibility of the co-migration of its derivative CYA [14]. Considering that the health risk caused by co-exposure to MEL and CYA is much higher than the exposure to a single substance [15], and considering the large population in China, it is important to explore the simultaneous migration of MEL and its derivatives from MEL-made tableware in China, as well as conduct a human risk assessment.

Currently, with the promotion of environmental protection concepts, the two kinds of tableware known as bamboo-made tableware and wheat straw-made tableware claim to be fully biodegradable and have gradually gained attention. However, despite the components of “bamboo fiber” and “wheat straw” occurring in the raw materials, their essence is still made of MEL–formaldehyde resin. During the manufacturing process of MEL–formaldehyde resin, additional bamboo fiber, corn starch, or wheat straw are incorporated. It is important to highlight that the incorporation of components like bamboo fiber may result in an increase in the intramolecular gaps within MEL–formaldehyde resins. This, in turn, could potentially intensify the migration of harmful components from such tableware. Currently, the foreign literature has substantiated the migration of MEL and formaldehyde from bamboo-made tableware, demonstrating a higher migration rate compared to that observed in MEL-made tableware [16,17]. However, the potential migration of MEL and its derivatives from bamboo- or wheat straw-made tableware has not been explored in China.

In this study, 46 pieces of tableware (bowls or cups) made of MEL, bamboo, and wheat straw were purchased randomly from Internet markets or retail shops in China. Meanwhile, four pieces of tableware made of glass or ceramic were used as the control group. We referred to the test conditions selected from the European Union Reference Laboratory for Food Contact Materials (EURL-FCM) [18]. The concentrations of MEL and its derivatives migrating from the tableware were quantified using an ultra-performance liquid chromatography–tandem mass spectrometry (UPLC–MS/MS) analysis. It combines the powerful separation capability of UPLC with the specific identification capability of MS, which has higher accuracy and precision than other technologies. The potential risks of exposure to MEL and its derivatives through contact with the tableware were assessed for both adults and infants.

## 2. Materials and Methods

### 2.1. Collection of Tableware

Between January and April 2022, 11 different brands of MEL-, bamboo-, wheat straw-made tableware (24 bowls and 22 cups) and 3 brands of glass- and ceramic-made tableware (2 bowls and 2 cups) were sampled randomly and purchased from Internet markets or retail shops in Shanghai, China. Other than the glass- and ceramic-made tableware, each brand of MEL/bamboo/wheat straw-made tableware we purchased had 3–5 duplicated samples. The detailed information of all tableware is shown in Table 1. The ceramic- and glass-made tableware items were purchased and used as the control group. The price of all tableware was about 2–16 China Yuan (CNY) per sample. To minimize experimental variability, the internal surfaces of all tableware items were gray or white, devoid of any pictorial adornments. The information regarding the internal surface area (ISA) of all tableware was calculated based on the inner diameter and depth per sample. All tableware items we purchased had a cylindrical shape for the purpose of facilitating the ISA calculation.

### 2.2. Chemicals

Analytical standard chemicals (MEL, ammeline (AMN), ammelide (AMD), CYA; purity ≥ 99%) and their corresponding isotopically labeled internal standards (IS; ^13^C_3_-MEL, ^13^C_3_-^15^N_3_-AMN, ^13^C_3_-^15^N_3_-AMD, ^13^C_3_-CYA; purity ≥ 99%) were purchased from Sigma-Aldrich (St. Louis, MO, USA) and Cambridge Isotope Laboratory (Waltham, MA, USA). Figure S1 presents the structures and CAS numbers of MEL and its three derivatives. The reagents used, including acetonitrile, formic acid, acetic acid, ammonium hydroxide, and methanol, were of HPLC-grade quality; however, hydrochloric acid and ammonium formate were of reagent-grade quality.

### 2.3. Migration Testing

The migration test conditions were adopted according to a dedicated guideline regarding food contact materials published by the EURL [18]. To test food contact materials that are used to hold hot beverages, the guideline recommends a migration temperature of 70 °C, migration time of 2 h, 3% acetic acid solution in water as a food simulant, and 3 repeated migration tests. The detailed operation processes were conducted as follows: each tableware was filled with the pre-warmed (70 °C) food simulant (3% acetic acid solution in water) up to 0.5 cm from the rim and immersed in water bath equipment at the designated temperature (70 °C) for 2 h. The tableware was covered with a glass lid during the two-hour migration test to prevent the gasification of liquid. After 2 h, the food simulant in each item of tableware was homogenized and transferred into a 15 mL centrifuge tube. After cooling, the simulant samples were stored in a −80 °C biobank refrigerator. After completing one experiment, all tableware underwent a simulated household washing process; the tableware items were washed thoroughly with distilled water three times, wiped gently with clean cotton textiles, and reused for the next experiment. Meanwhile, the glass- and ceramic-made tableware items were used as our blank groups and treated using the same conditions as the experimental groups. All tableware underwent three repeated migration tests.

### 2.4. Measurement of MEL and Its Derivatives

The detection methodology for the concentrations of MEL and its derivatives has been described in detail in previous studies [19,20]. In brief, the simulant (1 mL) mixed with IS mixtures (20 μL) was drawn into two centrifuge tubes and combined with 2 mL of hydrochloric acid (0.1 mol/L) or ammonia: water (10:90, *v*/*v*). Two mixtures were separately loaded into the activated CNW Poly-Sery Mixed Cation Exchange (MCX; 6 mL, 150 mg; ANPEL Lab. Tech. Inc., Shanghai, China) SPE column and CNW Poly-Sery Mixed Anion Exchange (MAX; 6 mL, 150 mg; ANPEL Lab. Tech. Inc., Shanghai, China) SPE column. Thereafter, the elution process was performed using 3 mL of ammonium hydroxide: methanol (5:95, *v*/*v*) or 3 mL of formic acid: methanol (2:98, *v*/*v*), followed by concentration to near dryness. Two residues were reconstituted with a 0.5 mL ammonium formate aqueous solution (5 mM/L, pH = 4): acetonitrile (5:95, *v*/*v*), and mixed and filtered through a 0.22 μm nylon filter for the UPLC-MS/MS analysis. The detailed UPLC-MS/MS parameters for four targeted analytes are presented in Table S1. A 2 μL analyte was injected into a Waters ACQUITY UPLC system combined with an AB Sciex API QTRAP 6500+ triple quadrupole mass spectrometer (AB Sciex, Waltham, MA, USA) for detection. The liquid chromatography column used was the Water ACQUITY UPLC BEH Amide column (1.7 μm, 2.1 × 100 mm; Milford, MA, USA). The mobile phase A solution was a 5 mM/L ammonium formate aqueous solution (pH = 4). The mobile phase B solution was an HPLC-grade acetonitrile solution. The separation chromatogram of MEL and its derivatives is shown in Figure S2.

### 2.5. Method Validation

A procedural blank (3% acetic acid solution) was assessed for each batch of 10 samples. No detectable levels of target analytes, including MEL and its derivatives, were observed in the blanks. The standard calibration curves of four substances were established using quantitative ions and the internal standard (IS) at concentrations ranging from 0.5 to 1000 ng/mL, exhibiting regression coefficients (R^2^) of > 0.99904 (Figure S3). The matrix sample (3% acetic acid solution) was mixed with a solution with a standard concentration to make the matrix-spiked samples, with concentrations of 0.2, 5, 50 and 200 ng/mL and 6 parallel samples for each concentration. The matrix-spiked samples were processed using the pretreatment method and then measured using UPLC-MS/MS. The average recoveries of the target compounds in the matrix-spiked samples ranged from 94.6 to 106.5%, with intra-day relative standard deviations (RSDs) in the range of 0.73–8.34%. The inter-day RSDs in the continuous 6-day experiments were less than 6.80%. The limits of detection (LOD) and quantitation (LOQ) were determined based on signal-to-noise ratios of 3 and 10, respectively (Table S2). LOD refers to the lowest amount that can be detected in a sample. LOQ refers to the lowest amount in a sample that can be quantitatively determined. The methodological parameters for the four target compounds are further elaborated in Table S3.

### 2.6. Risk Assessment

MEL/bamboo/wheat straw-made tableware items are extensively used as food containers to hold hot food for both adults and infants. Thus, the exposure risks of using these tableware items for adults and infants need to be evaluated urgently. We assumed that the food eaten by both adults and infants in a whole day was loaded in such tableware. Then, we conducted an external risk assessment by calculating the estimated daily intakes (EDIs) and hazard quotients (HQs) of MEL and its derivatives, respectively, based on the migration levels of MEL and its derivatives from such tableware, with two formulas adopted as follows. The HQ refers to the ratio of the dose of MEL and its derivatives ingested by humans using such tableware to the highest tolerable daily intake (TDI).

(1) EDI = C × FIR/BW

EDI (ng/kg bw/day) refers to the estimated daily intake of MEL and its derivatives in adults or infants using MEL/bamboo/wheat straw-made tableware as food containers. C (ng/mL) refers to the concentrations of MEL and its derivatives detected in the food simulant. FIR (mL) refers to the volume of estimated daily consumption by using the tableware, and BW (kg) indicates the estimated weight for the target population. For adults, the FIR was estimated to be 700 mL based on reports in the literature [7], and BW was calculated at 60 kg. For infants, the lower the body weight, the larger the EDI value. Thus, this study mainly assessed the EDI value for infants at 1 year of age. Based on a previous study, the FIR for 1-year-old infants (including all foods, water, and milk) was estimated to be 300 mL [16]. According to a Chinese survey on the physical growth and development of children under seven years of age published by the Capital Institute of Pediatrics in 2018, the average BW of infants aged 1 years old was estimated at 9.73 kg [21].

(2) HQ = EDI/TDI

TDI (ng/kg bw/day) refers to the highest tolerable daily intake of MEL and its derivatives for adults and infants. The potential risks of MEL exposure for adults and infants were assessed by the calculation of the HQ. So far, the TDI value for MEL has been recommended by several government organizations and toxicology studies. We chose three common TDI values for MEL exposure, 3150, 8100, and 63,000 ng/kg bw/day, suggested by Choi et al. [22], Hsieh et al. [23], and the US Food and Drug Administration [24], respectively. Since AMN and AMD have no set specific TDI values, the literature generally suggests that the TDI value for MEL can be used to substitute the TDI values for AMN and AMD [25]. The TDI value for CYA was assigned at 2500 ng/kg bw/day, which was recommended by Choi et al. [22]. Considering the realistic worst-case scenario, we calculated the HQ values to estimate the exposure risks for adults and 1-year-old infants based on the strictest TDI values. The TDI values for MEL, AMN, and AMD was recommended by Choi et al. [22] (3150 ng/kg bw/day), and the TDI value for CYA was recommended by Choi et al. [22] (2500 ng/kg bw/day). Meanwhile, the hazard index (HI) method was adopted to estimate the total exposure risk for MEL and its derivatives in adults and infants, that is, the accumulation of HQ values for MEL and its three derivatives. 

### 2.7. Statistical Analysis

Due to the technical limitations of UPLC-MS/MS, the detectable concentration of analyte was not considered to be zero even if the value was less than the corresponding LOD. The concentrations of MEL and its derivatives below the LOD values were replaced by the 1/√2 of LOD [26]. We measured the inner diameter and depth of each tableware item, which was used to calculate and estimate the volume (mL) of the simulant solution filled in the tableware during the migration tests. The concentrations (ng/mL) of MEL and its derivatives migrating from different tableware items (MEL/bamboo/wheat straw/glass/ceramic) were represented by mean and percentile values. The concentrations of MEL were corrected using the ISA based on the following formula (ng/cm^2^): concentrations (ng/mL) × estimated volume (mL)/ISA (cm^2^). The Shapiro–Wilk non-parametric test was employed to compare the differences in migration among the different tableware items from the same brand, as well as to compare the differences in the migration of MEL and its derivatives among the tableware items made of different materials. Pearson’s coefficient was used to calculate the correlations among the migration of MEL and its three derivatives. The Shapiro–Wilk non-parametric test was performed to explore the influence of basic characteristics regarding different tableware materials on the migration of MEL and CYA. Based on the mean or median values (inter-quartile range) of the migration of MEL and its derivatives, HQ values for adults and 1-year-old infants were calculated. The data analysis was performed using SPSS statistical software version 20.0, with two-tailed tests where <0.05 indicated statistical significance.

## 3. Results

As shown in Table S4, the total concentration of MEL and its derivatives (∑MEL) migrating from the different samples of the same brand had inconsistent results. Even if all *p* values were > 0.05, there was still a certain level of variation in the migration of ∑MEL among different samples from the same brand, with a 132-fold maximum concentration difference (sample 2 and 3 of brand 5). In addition, the results of our study indicated that a higher concentration of ∑MEL migrating from the tableware (sample 2 of brand 1) was observed when a crack occurred during the migration testing.

The concentrations of MEL and its derivatives migrating from different tableware materials are summarized in Table 2. Except for glass- or ceramic-made tableware, other tableware, including MEL-, bamboo-, and wheat straw-made tableware, exhibited MEL and its three derivatives migrating at different levels. MEL was the primary contributor to the ∑MEL concentration in MEL- and bamboo-made tableware, with an average concentration of 311.74 ng/mL and 687.68 ng/mL, respectively. The detection rates of both were more than 97% (the proportion of concentration above the LOD). A certain concentration of CYA was shown to migrate from MEL- and bamboo-made tableware. CYA was the primary contributor to the ∑MEL concentration in wheat straw-made tableware, with a detection rate of 61.11% and an average concentration of 23.40 ng/mL. Meanwhile a certain concentration of MEL was shown to migrate from wheat straw-made tableware. 

As the specific migration limit (SML) of MEL migrating from food-contacted tableware in China is 0.2 mg/dm^2^, we further corrected the MEL concentration migrating from different tableware materials based on their ISA (Table 3). After the ISA correction, the average MEL concentrations migrating from MEL-, bamboo- and wheat straw-made tableware were 0.07 mg/dm^2^, 0.17 mg/dm^2^ and <0.01 mg/dm^2^, respectively. The ratios of MEL concentrations migrating from MEL-, bamboo- and wheat straw-made tableware exceeding the SML in China were 2.33%, 35.90% and 0.00%, respectively. 

**Figure 1:**
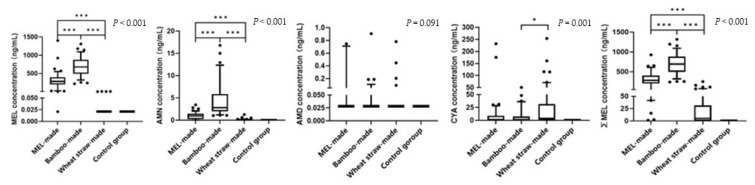
Comparison of the concentrations of MEL and its derivatives migrating from the different tableware materials (box diagram). Abbreviation: MEL, melamine; AMN, ammeline; AMD, ammelide; CYA, cyanuric acid; ∑MEL, the total concentrations of MEL and its derivatives. * *p* < 0.05, *** *p* < 0.001. Control group included glass- and ceramic-made tableware. In the box diagram, the box body indicates the median and inter-quartile range, the lower edge indicates P10, and the upper edge indicates P90.

Figure 1 illustrates the differences in the concentrations of MEL and its derivatives migrating from the different tableware materials. The results showed that the highest concentrations of ∑MEL, MEL, and AMN migrated from bamboo-made tableware, followed by MEL-made tableware, wheat straw-made tableware, and glass- or ceramic-made tableware (all *p* < 0.001). The order of CYA concentrations migrating from different tableware materials was as follows: wheat straw-made tableware > bamboo-made tableware > MEL-made tableware > glass- or ceramic-made tableware (*p* = 0.001). There was no significant difference in the AMD concentration migrating from the different tableware materials (*p* = 0.091).

Table S5 reveals the relationship between the concentrations of MEL and its three derivatives migrating from the different tableware materials. The concentration of MEL migrating from MEL-made, bamboo-made, and wheat straw-made tableware was positively correlated with the concentration of AMN, with correlation coefficients of 0.674, 0.816, and 0.287, respectively (all *p* < 0.05). No significant correlation was found among the concentrations of MEL, AMD, and CYA. We further analyzed the effect of the basic characteristics of the tableware on the migration of MEL and CYA, which is exhibited in Figure S4. Figure S4A shows the effect of repeated experiments on the migration of MEL and CYA. For MEL- and bamboo-made tableware, the migration of MEL was higher in the first migration, slightly decreased in the second migration, and increased again in the third migration (*p* > 0.05). For wheat straw-made tableware, the migration of CYA showed a gradual downward trend during three repeated experiments (*p* = 0.232). We also found that the migration of MEL in MEL-made and bamboo-made tableware purchased from retail shops was significantly higher than that of tableware purchased from Internet markets (*p* < 0.01) (Figure S4B). Moreover, the MEL concentration migrating from MEL-made tableware with a price of ≥10 CNY was higher than that of tableware with a price of <10 CNY (Figure S4C).

**Figure 2:**
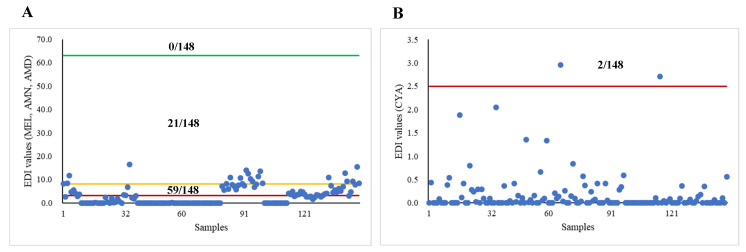
Calculation of EDI values for adults based on the concentration of MEL and its derivatives migrating from all tableware (*n* = 148). Abbreviation: MEL, melamine; AMN, ammeline; AMD, ammelide; CYA, cyanuric acid; EDI, estimated daily intakes. (**A**) Red, orange, and green lines represent TDI values recommended by Choi et al. [22] (3150 ng/kg bw/day), Hsieh et al. [23] (8100 ng/kg bw/day), and the US FDA [24] (63,000 ng/kg bw/day) for MEL exposure, respectively. (**B**) Red line represents TDI values recommended by Choi et al. [22] (2150 ng/kg bw/day) for CYA exposure. “*n*” represents the total number of experiments.

Figure 2 and Table 4 show the calculated EDI values and exposure risks for adults based on the concentrations of MEL and its derivatives migrating from different tableware materials. For the concentrations of MEL, AMN, and AMD, the EDI values exceeded the TDI values recommended by the US FDA [24], Hsieh et al. [23], and Choi et al. [22], by zero, twenty-one, and fifty-nine times, respectively. Based on the strictest TDI value, the proportions of the calculated HQ values ≥ 1 for MEL-, bamboo-, and wheat straw-made tableware were 53.50%, 92.30%, and 0.00%, respectively. For the CYA concentrations, the EDI values exceeded the TDI value recommended by Choi et al. [22] by two times. The proportions of the calculated HQ values ≥1 for MEL-, bamboo-, and wheat straw-made tableware were 2.30%, 0.00%, and 1.90%, respectively. 

**Figure 3:**
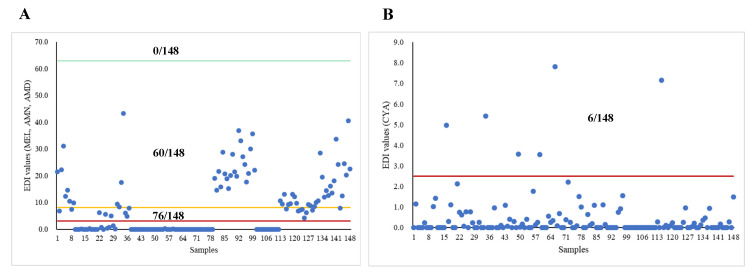
Calculation of EDI values for 1-year-old infants based on the concentration of MEL and its derivatives migrating from all tableware (*n* = 148). Abbreviation: MEL, melamine; AMN, ammeline; AMD, ammelide; CYA, cyanuric acid; EDI, estimated daily intakes. (**A**) Red, orange, and green lines represent TDI values recommended by Choi et al. [22] (3150 ng/kg bw/day), Hsieh et al. [23] (8100 ng/kg bw/day), and the US FDA [24] (63,000 ng/kg bw/day) for MEL exposure, respectively. (**B**) Red line represents TDI values recommended by Choi et al. [22] (2150 ng/kg bw/day) for CYA exposure. “*n*” represents the total number of experiments.

Figure 3 and Table 5 show the calculated EDI values and exposure risks for 1-year-old infants based on the concentrations of MEL and its derivatives migrating from different tableware materials. For the concentrations of MEL, AMN, and AMD, the EDI values exceeded the TDI values recommended by the US FDA [24], Hsieh et al. [23], and Choi et al. [22], by zero, sixty, and seventy-six times, respectively. Based on the strictest TDI value, the proportions of the calculated HQ values ≥ 1 for MEL-, bamboo-, and wheat straw-made tableware were 86.00%, 100.00%, and 0.00%, respectively. For the CYA concentrations, the EDI values exceeded the TDI value recommended by Choi et al. by six times [22]. The proportions of the calculated HQ values ≥1 for MEL-, bamboo-, and wheat straw-made tableware were 4.70%, 0.00%, and 7.40%, respectively. 

## 4. Discussion

This study explored the potential migration of MEL and its three derivatives from tableware made of MEL, bamboo, and wheat straw. The objective was to assess potential health risks for both adults and 1-year-old infants by simulating the typical usage of such tableware. The findings revealed that noticeable amounts of MEL and its derivatives were indeed migrating from tableware made of MEL, bamboo, and wheat straw. Furthermore, the risk assessment indicated that individuals, whether adults or infants, using such tableware may experience an elevated risk of exposure to MEL and its derivatives.

The study revealed a degree of variability in the total concentration migrating from different samples of the same brand, underscoring the inherent heterogeneity in raw materials even within products from an identical brand. This variability may be attributed to challenges in achieving fully condensed monomers, stemming from an inconsistent temperature or duration during the tableware manufacturing process. Similar observations have been documented in other studies [10,27,28]. Meanwhile, it was found that the concentrations of MEL and its derivatives migrating from the tableware increased significantly when a crack occurred during the migration tests. The results indicate that using tableware for containing food is not advisable in daily life when the items are cracked or broken.

In this study, MEL was the major substance migrating from MEL- and bamboo-made tableware. The study verified that MEL is the main raw material for manufacturing these tableware items. Under the same test conditions, the migration of MEL in this study was lower than that in other studies [7,11,12,29], probably because the items of tableware tested in different studies were manufactured in different years or different countries, which may affect the composition of the tableware and the migration of MEL. The results also showed that the MEL concentration migrating from the bamboo-made tableware was significantly higher than that from the MEL-made tableware (median 676.90 ng/mL vs. 281.85 ng/mL, *p* < 0.001). Meanwhile, Bouma et al. [16] found that the migration of formaldehyde from bamboo-made tableware was significantly higher than that from MEL-made tableware, indicating that MEL–formaldehyde resin tableware with added bamboo fibers may increase the migration of MEL and formaldehyde. One possible explanation is that the addition of bamboo fibers causes a structural alteration in the surface of the tableware, which accelerates the degradation of the polymer and leads to an increasing migration of formaldehyde and MEL. Meanwhile, the tableware made from MEL–formaldehyde resin and added bamboo fibers is more porous and increases the migration propensity.

To guarantee the safe use of food contact materials, the European Union has established a SML for MEL at 2.5 mg/kg [12,30]. The MEL concentrations migrating from all tableware in this study were in line with the SML of European Union. The Hygienic Standard for MEL–Formaldehyde Resin Products Used in Food Containers and Packaging Materials was promulgated in China in 2009 [31]. It stipulated that the SML of MEL for food contact materials in China was 0.2 mg/dm^2^. In this study, the average concentrations of MEL migrating from MEL- and bamboo-made tableware after correction were 0.07 mg/dm^2^ and 0.17 mg/dm^2^, respectively. The concentration of migration was similar to that in Wu et al.’s study [32], but lower than that in Bradley et al.’s study [27]. The proportions of MEL concentrations migrating from MEL- and bamboo-made tableware exceeding the SML in China were 2.33% and 35.90%, respectively, indicating that the production of some tableware did not meet the hygienic standards in China and should be examined by market supervision systems.

In addition to MEL, we found that its three derivatives could simultaneously migrate from MEL-, bamboo-, and wheat straw-made tableware, with CYA being the major substance migrating from wheat straw-made tableware. Most previous studies only examined the migration of MEL from MEL-made tableware, without considering the possible co-migration of its derivatives [5,7,10,27,33]. At present, the European Union and China have established SMLs of MEL for food contact materials, but the limits were established solely based on the adverse effect of MEL migrating from tableware on human health. This study revealed that the simultaneous migration of MEL and CYA was observed from such tableware. A number of human and animal studies have found that co-exposure to MEL and CYA resulted in higher nephrotoxicity and more serious renal damage than that from exposure to a single substance [34,35,36,37,38,39,40]. The dose of co-exposure to MEL and CYA without harmful effects is bound to be lower than the SMLs of MEL established by the European Union and China. Therefore, it is necessary to modify the SML of MEL for food contact materials and increase the SML of CYA simultaneously.

Currently, establishing a definitive safe threshold for MEL intake in humans proves challenging, and the potential health risks associated with prolonged exposure to low levels of MEL remain unclear. There have been several studies attempting to establish TDI values for assessing the exposure risk of MEL. The EFSA [41] established a TDI value of 0.2 mg/kg bw for MEL from the available toxicological data. The US FDA also recommended [24] a TDI value of 63,000 ng/kg bw/day for MEL based on animal toxicity experiments. The EDI values we calculated for adults and infants in this study did not exceed the two TDI values of the EFSA and US FDA. However, a study has found that a few children with MEL-associated urolithiasis were estimated to have consumed MEL at levels (0.04 to 62.67 mg/kg bw/day) below the TDI value recommended by the US FDA [42]. Therefore, Hsieh et al. and Choi et al. recommended reducing the TDI value of MEL to 8100 ng/kg bw/day and 3150 ng/kg bw/day [22,23]. Based on the above TDI values, this study estimated that the proportions of EDI values exceeding the two TDI values for adults were 21/148 and 59/148, respectively, while the proportions for 1-year-old infants were 60/148 and 76/148, respectively. These findings were consistent with previous studies; Chien et al. discovered that exposure to MEL through the use of MEL-made tableware by adults with an average weight of 60 kg resulted in an EDI value (0.75 mg) that surpassed the TDI value recommended by Hsieh et al. (0.48 mg) [7]. Similar results appeared in another study [11], which found that the level of MEL migrating from two types of cups from a day nursery was about 0.34 mg/dm^2^. Meanwhile, Choi et al. recommended a TDI value of 2500 ng/kg bw/day for CYA [23]; the proportions of EDI values exceeding the TDI value were 2/148 and 6/148 in adults and 1-year-old infants, respectively. In conclusion, the exposure risk of MEL and its derivatives through the usage of these tableware items were found to be higher in infants compared to adults, as evidenced by our findings.

Moreover, this study found that the use of bamboo-made tableware had the highest risk of exposure to MEL, AMN, and AMD for both adults and infants; wheat straw-made tableware had the highest risk of CYA exposure. Surprisingly, based on the strictest TDI value, the percentage of HQ values ≥1 for exposure to MEL and its three derivatives in 1-year-old infants was found to be as high as 100% when simulating food intake by bamboo-made tableware. The market of bamboo-made tableware primarily targets the parental demographic, with labels emphasizing its eco-friendly attributes, durability, impact resistance, and affordability. Some bamboo-made tableware is even labeled with prominent phrases such as “special bowl for children” and “special milk cup for children”, and the appearance is printed with cartoon characters to attract the attention of children or parents. Based on the findings of this study, it is recommended that parents should minimize or refrain from purchasing the bamboo- and other MEL-made tableware for serving hot food, which may increase the risk of suffering from MEL-related diseases in infants and young children.

There were some limitations present in this study. First, the concentrations of MEL and its derivatives migrating from the tableware would be influenced by various test conditions, such as temperature, food simulants, and repetitions [7]. In this study, the test conditions of the tableware only adopted the same standard of food contact materials recommended by the EURL, but it is imperative to conduct further migration tests under different conditions. Additionally, repeating an experiment with the same tableware did not keep the experimental conditions constant, which may influence the accuracy of the results. Second, the different tableware materials were identified by their product label or the product manual. The actual composition of the tableware we purchased had not been confirmed by laboratory detection methods. Third, we did not consider the surface finish of the samples, which could significantly change the real area of the materials’ exposure to the food simulant, and which could require an increase in the duration of the experiments.

## 5. Conclusions

In this study, we observed that varying concentrations of MEL and its three derivatives could migrate from tableware made of MEL, bamboo, and wheat straw. MEL was the primary contributor to ∑MEL concentrations in both MEL- and bamboo-made tableware, whereas CYA was found to be the main contributor in wheat straw-made tableware. It is concerning that some of the tested tableware made of MEL and bamboo did not meet the SML of MEL in China. Furthermore, the exposure assessment indicated that adults and 1-year-old infants may be at risk of exposure to MEL and its derivatives when using such tableware to hold hot food. Therefore, it is recommended that individuals should minimize or refrain from using tableware manufactured with MEL and bamboo fibers, so as to decrease the risk of developing MEL-related diseases.

## Figures and Tables

**Table 1:** Basic information of different tableware materials employed in the migration testing.

Sample Number	Brands	Labeled Material	Number of Samples	Price per Sample (CNY)	Shape	Purchase Channels	ISA (cm^2^)
1–3	Brand 1	MEL	3	5.06	Cup	IM	159.57
4–6	Brand 2	MEL	3	5.80	Bowl	IM	263.76
7–9	Brand 3	MEL	3	14.90	Cup	RS	168.56
10–12	Brand 4	MEL	3	15.90	Bowl	RS	193.42
13–15	Brand 3	MEL	3	10.90	Bowl	RS	227.65
16–19	Brand 5	Wheat straw	4	3.75	Bowl	IM	191.38
20–24	Brand 6	Wheat straw	5	2.00	Cup	IM	210.38
25–29	Brand 7	Wheat straw	5	2.00	Cup	IM	232.36
30–33	Brand 8	Wheat straw	4	3.50	Bowl	IM	305.05
34–36	Brand 9	Bamboo	3	8.30	Bowl	IM	171.82
37–39	Brand 10	Bamboo	3	7.30	Cup	IM	183.69
40–42	Brand 4	Bamboo	3	15.90	Cup	RS	127.80
43–46	Brand 11	Bamboo	4	7.90	Bowl	RS	180.55
47	Brand 12	Glass	1	9.90	Bowl	RS	95.46
48	Brand 11	Glass	1	14.90	Cup	RS	180.58
49	Brand 13	Ceramics	1	9.90	Bowl	RS	165.29
50	Brand 13	Ceramics	1	9.90	Cup	RS	178.60

Abbreviation: CNY, China Yuan; ISA, internal surface area; IM, internet markets; RS, retail shops.

**Table 2:** The concentrations of MEL and its derivatives migrating from the different tableware materials (*n* = 148).

		Concentration Range (ng/mL)	Detection Rate (%)	Mean (ng/mL)	Quantile Concentration (ng/mL)			
					P25	P50	P75	P95
MEL-made	MEL	<LOD–1402.35	97.67	311.74	196.94	281.85	392.32	863.41
tableware (*n* = 43)	AMN	<LOD–3.45	83.72	1.00	0.23	0.960	1.46	2.78
	AMD	<LOD–1.54	20.93	0.17	<LOD	<LOD	<LOD	1.27
	CYA	<LOD–231.99	48.840	15.20	<LOD	<LOD	8.82	146.63
Bamboo-made	MEL	217.79–1309.60	100.00	687.68	488.77	676.90	874.07	1181.07
tableware (*n* = 39)	AMN	1.05–16.80	100.00	4.36	1.98	2.79	5.85	15.01
	AMD	<LOD–0.91	17.95	0.06	<LOD	<LOD	<LOD	0.19
	CYA	<LOD–50.52	35.90	8.20	<LOD	<LOD	7.46	48.10
Wheat straw-made	MEL	<LOD–7.56	7.41	0.35	<LOD	<LOD	<LOD	3.92
tableware (*n* = 54)	AMN	<LOD–1.27	25.93	0.10	<LOD	<LOD	0.07	0.54
	AMD	<LOD–0.78	7.41	0.05	<LOD	<LOD	<LOD	0.26
	CYA	<LOD–253.70	61.11	23.40	<LOD	4.00	31.33	127.11
Other tableware ^a^	MEL	<LOD	0.00	<LOD	<LOD	<LOD	<LOD	<LOD
(*n* = 12)	AMN	<LOD	0.00	<LOD	<LOD	<LOD	<LOD	<LOD
	AMD	<LOD	0.00	<LOD	<LOD	<LOD	<LOD	<LOD
	CYA	<LOD	0.00	<LOD	<LOD	<LOD	<LOD	<LOD

Abbreviation: MEL, melamine; AMN, ammeline; AMD, ammelide; CYA, cyanuric acid. ^a^ Other tableware included glass- and ceramic-made tableware. “*n*” represents the total number of experiments.

**Table 3:** The ISA-corrected concentrations of MEL migrating from the different tableware materials (*n* = 148).

	Mean of MEL (mg/dm^2^)	Quantile Concentration of MEL (mg/dm^2^)				Non-Compliant Rate (%)
		P25	P50	P75	P95	
MEL-made tableware (*n* = 43)	0.07	0.05	0.07	0.09	1.14	2.33
Bamboo-made tableware (*n* = 39)	0.17	0.10	0.15	0.22	0.36	35.90
Wheat straw-made tableware (*n* = 54)	<0.01	<0.01	<0.01	<0.01	<0.01	0.00
Other tableware ^a^ (*n* = 12)	<0.01	<0.01	<0.01	<0.01	<0.01	0.00

Abbreviation: MEL, melamine; ISA, internal surface area. ^a^ Other tableware included glass- and ceramic-made tableware. The non-compliant rate (%) was calculated as the percentage of the MEL concentration > 0.2 mg/dm^2^ based on the SML in China for the food contact materials. “*n*” represents the total number of experiments.

**Table 4:** Risk assessment for exposure to MEL and its derivatives in adults based on the concentrations migrating from different tableware materials.

		HQ Value				Percentage (HQ ≥ 1, %)
		Mean	Median	P25	P75	
MEL-made	MEL + AMN + AMD	1.16	1.05	0.73	1.46	53.50
tableware (*n* = 43)	CYA	0.07	<0.01	<0.01	0.04	2.30
	∑MEL	1.23	1.06	0.78	1.52	53.50
Bamboo-made	MEL + AMN + AMD	2.56	2.51	1.82	3.26	92.30
tableware (*n* = 39)	CYA	0.04	<0.01	<0.01	0.04	0.00
	∑MEL	2.60	2.58	1.86	3.2	92.30
Wheat straw-made	MEL + AMN + AMD	<0.01	<0.01	<0.01	<0.01	0.00
tableware (*n* = 54)	CYA	0.11	0.02	<0.01	0.15	1.90
	∑MEL	0.11	0.02	<0.01	0.15	1.90
Other tableware ^a^	MEL + AMN + AMD	<0.01	<0.01	<0.01	<0.01	0.00
(*n* = 12)	CYA	<0.01	<0.01	<0.01	<0.01	0.00
	∑MEL	<0.01	<0.01	<0.01	<0.01	0.00

Abbreviation: MEL, melamine; AMN, ammeline; AMD, ammelide; CYA, cyanuric acid; ∑MEL, the total concentrations of MEL and its derivatives; HQ, hazard quotients. ^a^ Other tableware included glass- and ceramic-made tableware. In HQ calculation, the TDI value was acquired using the strictest standard based on the current studies. The TDI value of MEL, AMN, and AMD was recommended by Choi et al. [22] (3150 ng/kg bw/day), and the TDI value of CYA was recommended by Choi et al. [22] (2500 ng/kg bw/day). “*n*” represents the total number of experiments.

**Table 5:** Risk assessment for exposure to MEL and its derivatives in 1-year-old infants based on the concentrations migrating from different tableware materials.

		HQ Value				Percentage (HQ ≥ 1, %)
		Mean	Median	P25	P75	
MEL-made	MEL + AMN + AMD	3.06	2.78	1.94	3.85	86.00
tableware (*n* = 43)	CYA	0.19	<0.01	<0.01	0.11	4.70
	∑MEL	3.25	2.79	2.06	4.01	86.00
Bamboo-made	MEL + AMN + AMD	6.77	6.64	4.81	8.62	100.00
tableware (*n* = 39)	CYA	0.10	<0.01	<0.01	0.09	0.00
	∑MEL	6.88	6.81	4.92	8.62	100.00
Wheat straw-made	MEL + AMN + AMD	0.01	<0.01	<0.01	<0.01	0.00
tableware (*n* = 54)	CYA	0.29	0.05	<0.01	0.39	7.40
	∑MEL	0.29	0.06	<0.01	0.39	7.40
Other tableware ^a^	MEL + AMN + AMD	<0.01	<0.01	<0.01	<0.01	0.00
(*n* = 12)	CYA	<0.01	<0.01	<0.01	<0.01	0.00
	∑MEL	<0.01	<0.01	<0.01	<0.01	0.00

Abbreviation: MEL, melamine; AMN, ammeline; AMD, ammelide; CYA, cyanuric acid; ∑MEL, the total concentrations of MEL and its derivatives; HQ, hazard quotients. ^a^ Other tableware included glass- and ceramic-made tableware. In HQ calculation, the TDI value was acquired using the strictest standard based on the current studies. The TDI value of MEL, AMN and AMD was recommended by Choi et al. [22] (3150 ng/kg bw/day), and the TDI value of CYA was recommended by Choi et al. [22] (2500 ng/kg bw/day). “*n*” represents the total number of experiments.

## Data Availability

The datasets of this study are available from the corresponding author on reasonable request.

## Supplementary Materials

The following supporting information can be downloaded at: https://www.mdpi.com/article/10.3390/toxics12020143/s1. Figure S1: The structures and CAS numbers of MEL and its derivatives; Table S1: The MRM scan model and the mass spectrometry conditions of UPLC-MS/MS for MEL and its derivatives; Figure S2: Separation chromatogram of MEL and its derivatives (standard concentration of 20 ng/mL); Figure S3: Calibration curves of MEL and its derivatives (concentration range 0.5–1000 ng/mL); Table S2: The retention time and the limit of detection of the detected method for MEL and its derivatives; Table S3: The recoveries and the relative standard deviations of the detected method for MEL and its derivatives; Table S4: Comparison of the total concentrations of MEL and its derivatives migrated from the different samples of the same brand (ng/mL); Table S5: Correlations of the concentrations of MEL and its derivatives migrated from different materials-made tableware; Figure S4: Effects of basic characteristics of the different materials-made tableware on the migrating concentrations of MEL and CYA (A–C).
