# Supplementary material for: Migration of Melamine and Its Derivatives from Melamine/Bamboo/Wheat Straw-Made Tableware Purchased from Internet Markets or Retail Shops in China
Source: Toxics. 2024 Feb 9;12(2):143. doi: 10.3390/toxics12020143 (PMC10892781; doi:10.3390/toxics12020143)
Supplement: Supplementary file 1 [file toxics-12-00143-s001.zip › toxics-2816542-supplementary.pdf]

Supplemental Materials:

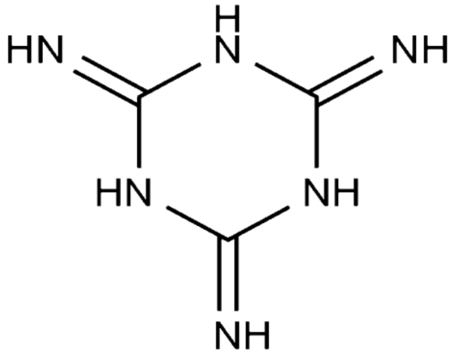

Melamine (MEL), CAS: 108-78-1

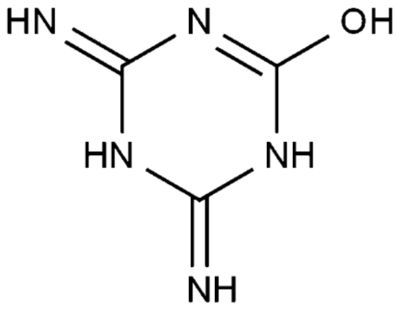

Ammeline (AMN), CAS: 645-92-1

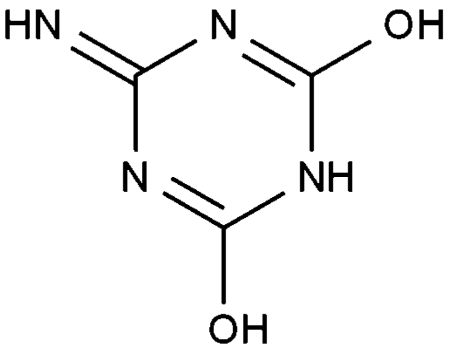

Ammelide (AMD), CAS: 645-93-2

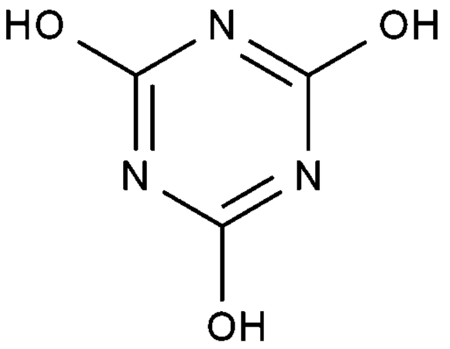

Cyanuric acid (CYA), CAS: 108-80-5

Figure S1. The structures and CAS numbers of MEL and its derivatives

Table S1. The MRM scan model and the mass spectrometry conditions of UPLC-MS/MS for MEL and its derivatives

Chemistries	Ion pair (m/z)	ESI	DP/V	EP/V	CE/V	ECP/V
MEL	127.1/85.0*	+	99	5	25	21
	127.1/68.0	+	40	3	40	17
AMN	128.1/86.0*	+	61	10	16	14
	128.1/69.0	+	101	4	33	31
AMD	126.8/84.0*	-	-19	-8	-16	-9
	126.8/42.0	-	-21	-4	-38	-6
CYA	127.9/42.0*	-	-21	-9	-30	-15
	127.9/85.0	-	-59	-10	-14	-20
$^{13}C_3$, $^{15}N_3$-MEL	133.1/89.0*	+	99	5	25	21
	133.1/72.0	+	40	3	40	17
$^{13}C_3$-AMN	131.1/88.0*	+	61	10	16	14
	131.1/71.0	+	101	4	33	31
$^{13}C_3$-AMD	129.9/86.0*	-	-19	-8	-16	-9
	129.9/43.0	-	-21	-4	-38	-6
$^{13}C_3$, $^{15}N_3$-CYA	133.9/44.0*	-	-21	-9	-30	-15
	133.9/89.0	-	-59	-10	-14	-20

Abbreviation: UPLC-MS/MS, ultra-performance liquid chromatography-tandem mass

spectrometry; MEL, melamine; AMN, ammeline; AMD, ammelide; CYA, cyanuric acid; MRM, multiple reaction monitoring; ESI, electrospray ionization; DP, declustering potential; EP, enrollment potential; CE, collision energy; ECP, emission energy potential. The first ion was precursor ion, and the second ion was product ion in ion pair. *Quantitative ion.

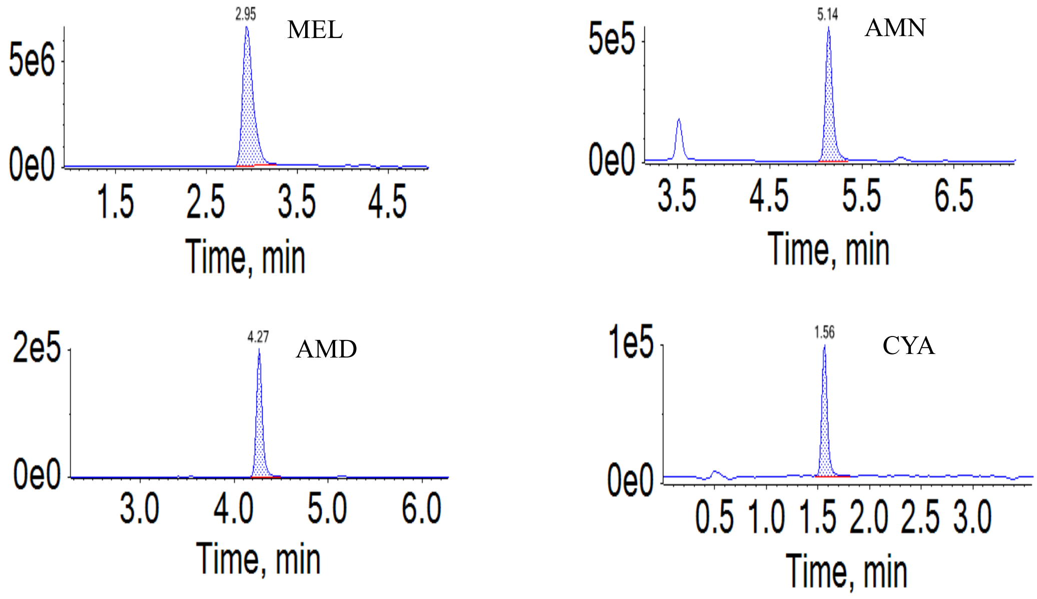

Figure S2. Separation chromatogram of MEL and its derivatives (standard concentration of 20 ng/mL)

Abbreviation: MEL, melamine; AMN, ammeline; AMD, ammelide; CYA, cyanuric acid.

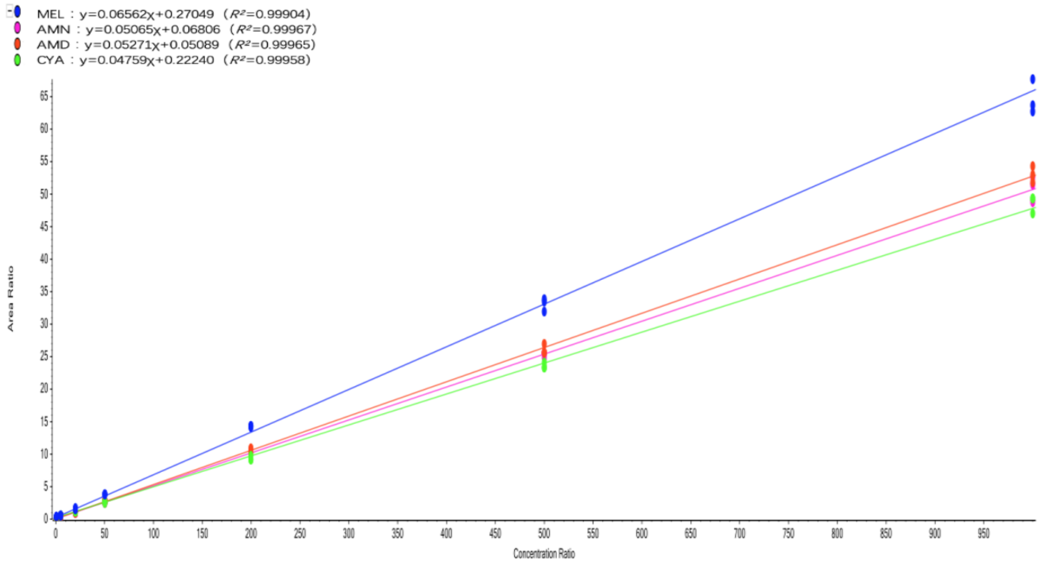

Figure S3. Calibration curves of MEL and its derivatives (concentration range

0.5-1000 ng/mL)

Abbreviation: MEL, melamine; AMN, ammeline; AMD, ammelide; CYA, cyanuric acid.

Table S2. The retention time and the limit of detection of the detected method for MEL and its derivatives

Chemistries	RT (min)	LOD (ng/mL)	LOQ (ng/mL)
MEL	2.95	0.03	0.11
AMN	5.14	0.04	0.12
AMD	4.27	0.04	0.14
CYA	1.56	0.05	0.15

Abbreviation: RT, retention time in minutes; LOD, limit of detection; LOQ, limit of quantitation; MEL, melamine; AMN, ammeline; AMD, ammelide; CYA, cyanuric acid.

Table S3. The recoveries and the relative standard deviations of the detected method for MEL and its derivatives

Chemistries	Standard concentrations	Intra-day of RSD (%)	Recoveries (%)	Inter-day of RSD (%)
MEL	0.2 ng/mL	8.03	102.66	3.80
	5 ng/mL	6.88	94.99	3.62
	50 ng/mL	2.10	106.51	2.22
	200 ng/mL	0.73	98.50	1.38
AMN	0.2 ng/mL	8.34	95.50	6.15
	5 ng/mL	6.93	100.36	5.29
	50 ng/mL	2.81	99.53	3.17
	200 ng/mL	0.75	100.11	2.13
AMD	0.2 ng/mL	7.44	99.27	4.71
	5 ng/mL	5.86	99.34	1.76
	50 ng/mL	2.29	100.86	1.20
	200 ng/mL	1.80	99.80	0.80
CYA	0.2 ng/mL	7.66	94.55	6.80
	5 ng/mL	6.65	99.09	6.70
	50 ng/mL	6.28	100.88	4.47
	200 ng/mL	5.91	99.86	2.99

Abbreviation: RSD, relative standard deviations; MEL, melamine; AMN, ammeline; AMD, ammelide; CYA, cyanuric acid.

Table S4. Comparison of the total concentrations of MEL and its derivatives migrated from the different samples of the same brand (ng/mL)

Number	Brand	Sample 1	Sample 2	Sample 3	Sample 4	Sample 5	*P* value
1	Brand 1	312.07	1580.03*	197.79			0.076
2	Brand 2	41.61	50.50	41.58			0.733
3	Brand 3	346.78	468.66	439.56			0.733

4	Brand 4	355.69	302.53	401.08			0.113
5	Brand 3	229.95	233.07	283.43			0.393
6	Brand 5	33.76	0.27	35.64	0.42		0.319
7	Brand 6	11.87	0.29	12.32	0.18	32.57	0.347
8	Brand 7	<LOD	3.94	2.12	9.48	1.06	0.754
9	Brand 8	7.65	8.19	<LOD	8.40		0.885
10	Brand 9	696.92	481.32	318.96			0.177
11	Brand 10	617.88	671.77	611.27			0.670
12	Brand 4	790.68	405.14	777.19			0.430
13	Brand 11	695.37	1074.77	728.50	972.81		0.147
14	Brand 12	<LOD					-
15	Brand 11	<LOD					-
16	Brand 13	<LOD					-
17	Brand 13	<LOD					-

There were three repeated experiments for all samples, and the data was shown as median value. Shapiro-Wilks non-parametric test was used to compare the difference of the total concentrations of MEL and its derivatives migrated from different samples of the same brand. *The sample occurred a crack during the first experiment. Number 14-17 were the tableware made of glass and ceramic as the control group.

Table S5. Correlations of the concentrations of MEL and its derivatives migrated from different materials-made tableware

		MEL	AMN	AMD	CYA
MEL-made	MEL	1.000	0.674**	-0.205	0.056
tableware	AMN	0.674**	1.000	-0.133	0.108
	AMD	-0.205	-0.133	1.000	0.161
	CYA	0.056	0.108	0.161	1.000
Bamboo-made	MEL	1.000	0.816**	0.090	-0.099
tableware	AMN	0.816**	1.000	0.283	-0.290
	AMD	0.090	0.283	1.000	-0.127
	CYA	-0.099	-0.290	-0.127	1.000
Wheat straw-made	MEL	1.000	0.287*	-0.080	-0.199
tableware	AMN	0.287*	1.000	0.043	0.108
	AMD	-0.080	0.043	1.000	0.003
	CYA	-0.199	0.108	0.003	1.000

Abbreviation: MEL, melamine; AMN, ammeline; AMD, ammelide; CYA, cyanuric acid. *P<0.05，**P<0.01.

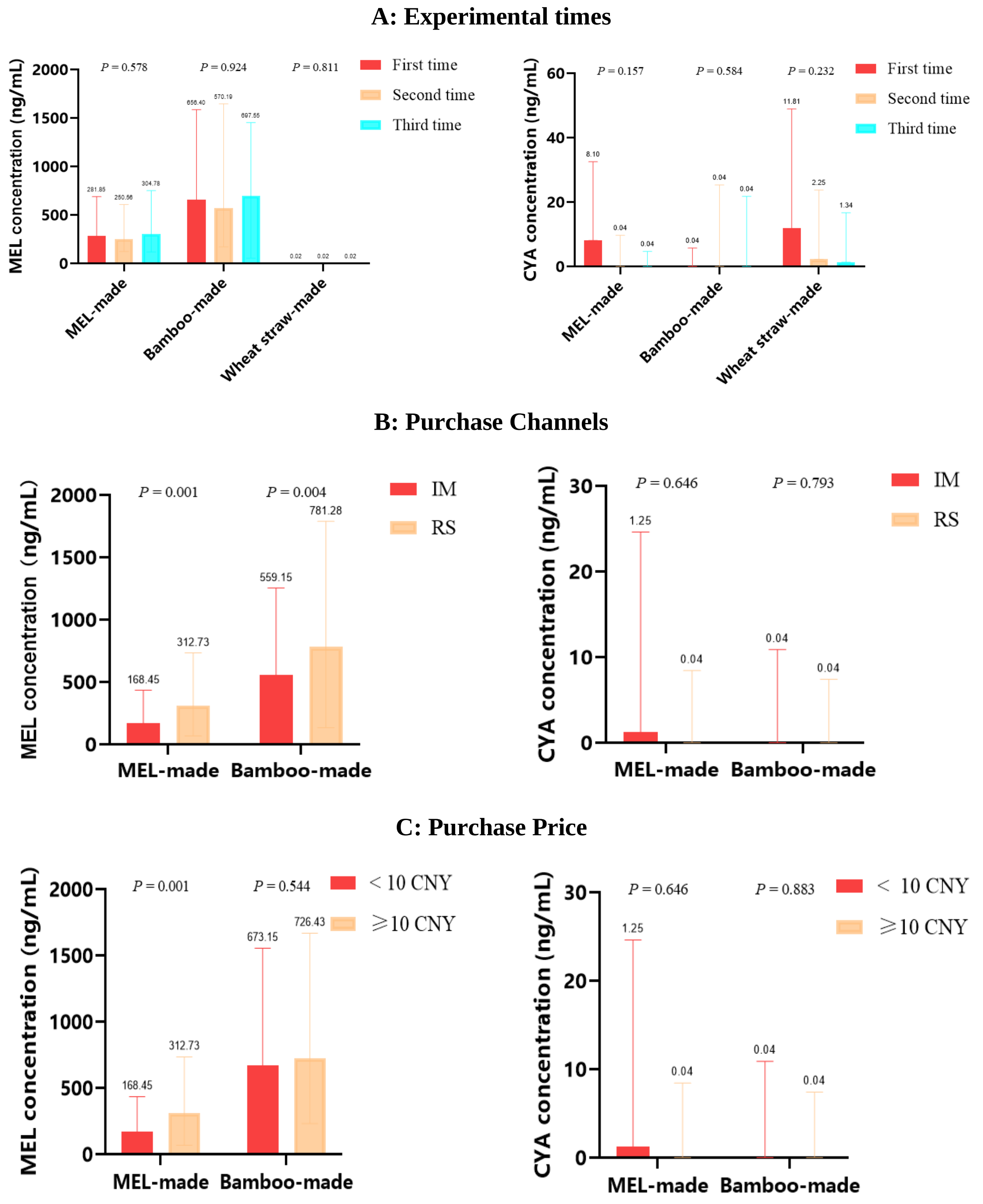

Figure S4. Effects of basic characteristics of the different materials-made tableware on the migrating concentrations of MEL and CYA (A-C)

Abbreviation: MEL, melamine; CYA, cyanuric acid; IM, internet markets; RS, retail shops; CNY, China Yuan. The data was shown as the median and inter-quartile range. A: Effect of experimental times on the concentrations of MEL and CYA migrated from the different materials-made tableware; B: Effect of purchase pathways on the concentrations of MEL and CYA migrated from the different materials-made tableware; C: Effect of purchase price on the concentrations of MEL and CYA migrated from the different materials-made tableware. All wheat straw-made tableware was purchased from the internet markets whose price was less than 10 CNY, so that the analysis of purchase channel (B) and purchase price (C) was not included.
